# Effects of WAKE.TAIWAN Healthy Lifestyle Web-Based Promotion Programs on Adults’ Awareness of Self-perceived Weight Status and Their Healthy Lifestyle Stages: Retrospective Analysis

**DOI:** 10.2196/41944

**Published:** 2023-05-25

**Authors:** Asta Y Z Lord, Wen-Harn Pan

**Affiliations:** 1 Institute of Population Health Sciences National Health Research Institutes Zhunan Town, Miaoli County Taiwan; 2 Institute of Biomedical Sciences Academia Sinica Taipei Taiwan

**Keywords:** healthy eating, active living, obese, weight, social media, website, web based, health behavior, online health promotion, obesity, chronic disease, healthy lifestyle, lifestyle, health promotion, health education, online health information, quasi-experimental, questionnaire, survey research, applied method, nutrition, food, eat, confidence interval, generalized linear model

## Abstract

**Background:**

Obesity is a major risk factor of many chronic diseases. However, current obesity control policies and actions are not sufficient to halt the pandemic. It has been documented that more than half of all adults are not able to interpret their own weight status, not to mention to practice healthy lifestyles. Social media and interactive websites can reach people on a long-term basis, which may be used as intervention vehicles to build up cognition for weight control and to promote healthy behavior practices.

**Objective:**

WAKE.TAIWAN is an ongoing web-based multifaceted healthy lifestyle promotion program with social media and interactive websites as the intervention vehicle. This study aimed to examine whether adults reached by our program would have increased awareness to their own anthropometric measures, correctly judge their body weight status, and practice healthy behaviors over time.

**Methods:**

This study adopted a quasi-experimental design with web-based questionnaire surveys. The experimental group consisted of WAKE.TAIWAN Facebook group members aged 20-65 years who have used the interactive website health education resources (n=177). The group was further stratified into 2 subgroups based on their duration of participation (E1 group: duration <1 year; E2 group: duration ≥1 year). The control group consisted of other Facebook users (n=545) in the same age range who had not been exposed to the health education materials of this project. A total of 722 people (male: n=267, 37%; and female: n=455, 63%) participated in our survey in 2019. Data were analyzed to evaluate program effectiveness using a generalized linear model.

**Results:**

The proportion of people correctly interpreting their own weight status in the experimental group was greater than that of the control group (control group: 320/545, 58.7%; group E1: 53/88, 60%; and group E2: 64/89, 72%). The E2 experimental group was significantly better than the control group in paying attention to weight-related measures and in correctly interpreting their own weight status (odds ratio 1.73, 95% CI 1.04-2.89; *P*=.04). With respect to the behavioral stages of practicing healthy eating and active living, both experimental groups, E1 and E2, performed significantly better than the control group (group E1: *P*=.003 and *P*=.02; and group E2: *P*=.004 and *P*<.001, respectively).

**Conclusions:**

This study demonstrates that the longer the participants were exposed to our social media–based programs, the higher the proportion of them that would have the correct judgement on their weight status and fall in the higher stages of healthy lifestyle behaviors. A longitudinal follow-up survey is in place to verify these findings.

## Introduction

Overweight and obesity are not only known as important causative factors for various chronic diseases and cancers [1-8]; recent epidemiological studies of COVID-19 also found that adults who are overweight and obese are more likely to develop severe adverse outcomes than their normal weight counterparts [9-14]. Although research evidence strongly supports that obesity control and prevention is a crucial means to promote population health, and it has been a health policy priority by the World Health Organization for a long time, the prevalence of obesity has not yet been controlled by existing health policies and actions worldwide [15-18]. Taiwan is not an exception. According to recent findings from the National Nutrition and Health Survey in Taiwan, the prevalence and severity of obesity are on the rise [19,20]. Apparently, a large number of adults have not been reached or influenced by current policy actions.

Several large surveys have pointed out that about 50% of adults underestimated their weight status [21], whereas the rate could reach as high as 40% to 70% in adults who are overweight or obese [22,23]. According to a recent study [24], adults who are overweight or obese who could correctly interpret their weight status were more willing to control their weight than those who could not. In other words, it is more likely for people to consider weight control when they know that their weight is beyond the healthy range. Therefore, the ability to correctly interpret one’s own weight status may be a key to reaching or maintaining a healthy weight.

In addition, efficacy studies of weight control interventions in the literature have found that most people who successfully lose weight during the intervention often regain weight after the intervention ends, and only about 20% to 30% of them maintain their reduced weight [25,26]. Literature also points out that influential factors for long-term weight control are multidimensional. It has been gradually recognized that an effective interventional design for weight control or maintenance requires sufficient internal and external efforts and supports, such as continuous self-monitoring and goal setting; staying up-to-date with current knowledge; social support; group interaction; and reinforcement from improved health check-up results, nicer physical appearance, etc [25,27-31]. More and more intervention studies on weight control and health behavior promotion used interactive websites as platforms since they allow timely knowledge update and include web-based communities as an interaction medium for building social supports and encouragement [32-37]. However, such interventions have rarely lasted for more than one year. The effect of the real-world long-term intervention through web-based vehicles needs to be explored. Therefore, this study developed multifaceted strategies and constructed an interactive environment (ie, an open web-based community and interactive websites with learning materials) to support long-term intervention and to verify that such design can effectively strengthen participants’ attention on body weight–related measures; improve their ability to correctly interpret weight status; increase the likelihood of adopting and maintaining healthy behaviors; and eventually improve weight status, body composition, and images.

This study anticipated that those who have been exposed to health education information and participated in the designed activities of web-based communities would improve knowledge and cognition on healthy body weight, attend to and understand relevant anthropometric indicators (such as waist circumference and body fat percentage), and practice and maintain healthy lifestyles.

## Methods

### Web-Based Environment Settings and Intervention Design

The web-based environment settings of this study emphasized “Easy Access” and “Interactive Learning.” There were 2 major components, “WAKE.TAIWAN Learning Group on Facebook” and “WAKE.TAIWAN Healthy Lifestyle Website,” which functioned as the main intervention channel and interactive learning website, respectively.

The multifaceted strategies were designed as interventions and are described below:

- Provide “step-by-step” conceptual knowledge for building a healthy body: By applying the “Group Guides” function of the learning group interface, we provide participants with basic concepts on healthy eating and effective exercise. Each post in the Group Guides also has a “category label” to help group members find relevant posts, hyperlink resources, and questions and answers easily. Through interactive responses to posts and questions and answers, participants can share their successful and fun personal experiences of healthy living, which encourages more interaction with other group members and helps to deepen their understanding of weight control knowledge and practice healthy lifestyles with fun.
- Continuously provide comprehensive learning materials and information on weight control and healthy lifestyles through designed platform: The WAKE.TAIWAN Healthy Lifestyle website was established as the primary platform for learning resources, providing well-organized and up-to-date evidence-based materials and information. This includes knowledge on healthy eating; anthropometric parameters (such as BMI, body fat percentage, muscle mass, and waist circumference); effective exercise; and risk evaluation tools for diabetes, hypertension, and coronary heart disease. Audiovisual materials are presented on the WAKE.TAIWAN YouTube channel, which is hyperlinked to this website. The channel provides exercise videos with varying levels of intensity that are designed for different occasions. It also offers educational videos to address common myths about healthy eating. Participants can access the learning information through posts on the WAKE.TAIWAN Learning Group on Facebook or by visiting the WAKE.TAIWAN Healthy Lifestyle website and YouTube channel directly. In addition, the website, resources, and activities are open to the general public.
- Periodically provide activities with rewards to encourage long-term participation and practice health behavior: Reward activities are one of the key intervention strategies in this study. The main purpose of the reward activities is to attract participants to keep interacting with learning group members, sharing their thoughts and actions on building a healthy body, and frequently visiting the website for health information and to practice the recommended healthy lifestyle activities. At the same time, the activity design encourages participants to bring friends to join and share their experiences practicing the recommended healthy lifestyle activities.

### Web-Based Survey for Effectiveness Evaluation

#### Questionnaire Items

The main questionnaire items included sex, age, education level, employment status, working hours, the history of chronic diseases, height, weight, waist circumference, body fat percentage, self-classified BMI status, the stage of healthy eating behaviors (HEB), the stage of active living behaviors (ALB), and the use of health resources.

The measurements of concern in this study included the attention to one’s waist circumstance and body fat percentage, the correctness of self-perceived body weight, the use of health resources, and the stages of HEB and ALB. To assess performance of HEB and ALB, we applied the Stage of Change Model for defining behavioral stages. Respondents were asked to select the options that matched their behavioral performance. The 5 stages are defined as follows. Stage 1 is the precontemplation period (scored as 1 point): “I have not realized that this is an important matter”; stage 2 is the contemplation period (scored as 2 points): “I have realized that it is an important thing, but I have not started thinking about how to do it”; stage 3 is the preparation period (scored as 3 points): “I have realized that it is an important thing, and I have begun to think about how to do it”; stage 4 is the action period (scored as 4 points): “I have realized that it is an important thing, and I have begun to practice it in the past six months”; and stage 5 is the maintenance period (scored as 5 points): “I have maintained it for over half year and continue to implement it.” In addition, we added another option: “I used to practice this behavior, but it was suspended for some reason.” This type of respondents was further inquired about the reason for the suspension, and they were not included in the comparison between the experimental group and the control group.

#### Questionnaire Validity

To examine the validity of the questionnaire, 5 domain experts were invited to score the content validity index (CVI). Both the item-level CVI (I-CVI) and the universal agreement scale-level CVI (S-CVI/UA) were applied to examine the quality of “wording explicitness” and “content applicability” [38-41]. The scoring scale was rated from 1 to 4, where 1=not relevant, 2=somewhat relevant, 3=quite relevant, and 4=highly relevant.

The I-CVI was calculated by dividing the number of experts who rated the item as relevant (a score of 3 or 4) by the total number of experts who rated the item [42]. A value of 1 indicates that all experts rated the item as relevant. Since all items of the questionnaire were rated between 3 and 4, the I-CVI was equal to 1. Having this I-CVI value for the questionnaire is concordant with the advised level while the number of experts was 5 or fewer. This means that in this study, unanimous agreement among experts indicated that the wording of the item was concise and applicable, which helps obtain valid data suitable for this survey.

The S-CVI/UA was calculated by determining the proportion of items that all experts rated as relevant (a score of 3 or 4) [42]. A value greater than 0.80 is generally considered to indicate good content validity for the entire questionnaire. In this study, all items achieved a score of 3 or 4 by all experts in the rating panel, resulting in an S-CVI/UA value of 1, which is consistent with the recommended level of >0.80.

To eliminate unreasonable answers to the questionnaire, the numerical question items were checked with a rational range set by the system. The rationality of the answer between questions were examined by researchers.

### Ethics Approval

This study conducted a survey using a web-based anonymous questionnaire, without collecting personally identifiable information such as name and full ID number. Therefore, the questionnaire data cannot be linked to individual identities. Prior to conducting the survey, the research plan and questionnaire content were submitted for review and approval by the institutional review board committee of the National Health Research Institutes (IRB_EC1070802_E), and participants’ consent forms were waived.

### Participants Recruitment and Data Collecting Procedures

Participants in this study were adults aged 20 to 65 years. The participants in the experiment group were recruited from the WAKE.TAIWAN Facebook learning group. The Facebook group was established by WAKE.TAIWAN research team for promoting healthy lifestyles and weight control. Based on the duration of participation in the WAKE.TAIWAN Facebook learning group, participants who were exposed to the health intervention and had participated in web-based activities for less than 1 year were classified into group E1. Those who had participated and been exposed for 1 year or more were assigned into group E2. To control for intervention conditions and reduce background bias in questionnaire surveys, the control group was set up as Taiwan Facebook users who had never been exposed to any intervention information from this study.

The questionnaire for the experimental group was distributed through the WAKE.TAIWAN Facebook learning group, whereas for the control group, it was sent via a Facebook group created under the name of a local township. The questions in the grouping section aimed to gather information on the survey participants’ familiarity with the web-based WAKE.TAIWAN system. In the initial part of the survey, participants were categorized as either belonging to the experimental group (those who had been exposed) or the control group (those who had not been exposed) based on their responses to these questions. They were then directed to the respective questionnaires for the experimental and control groups.

A total of 800 questionnaires were collected within 3 days after the survey was launched. After excluding invalid or repetitive records and those with extreme values, 722 valid questionnaires were obtained.

### Statistical Analysis

Logic check for rational numerical range was performed to eliminate unreasonable answers to the questions. Statistical measures such as the number of participants, percentages, mean, and 95% CI were used to describe the participant characteristics. A generalized linear model was used to estimate the odds ratio (OR) or to test the differences of each measure between the groups. All the data management and analyses were conducted by using the statistical software SPSS (version 27; IBM Corp).

## Results

### Samples Characteristics

Participants’ descriptive statistics are shown in Table 1. The proportion of female participants (455/722, 63%) was higher than that of male participants (267/722, 37%). The participants were aged 38 years on average, and many (321/722, 44.5%) were aged within the range of 20 to 35 years. Over half of these participants (389/722, 53.9%) were college graduates. A small fraction (65/722, 9%) of the participants reported a history of chronic diseases. Furthermore, 38.2% (276/722) of the participants had a BMI over 24 kg/m^2^.

**Table 1:** Participant characteristics (n=722).

Characteristics and variable items			Control (n=545)		E1^a^ (n=88)		E2^b^ (n=89)		Total (n=722)
**Sex, n (%)**									
	Male	194 (35.6)		42 (47.7)		31 (34.8)		267 (37)	
	Female	351 (64.4)		46 (52.3)		58 (65.2)		455 (63)	
**Age group (years), n (%)**									
	20-35	272 (49.9)		26 (29.5)		23 (25.8)		321 (44.5)	
	36-45	125 (22.9)		41 (46.6)		39 (43.8)		205 (28.4)	
	46-65	148 (27.2)		21 (23.9)		27 (30.3)		196 (27.1)	
**Age (years) mean (95% CI)**			37.6 (36.6-38.5)		39.7 (37.5-41.8)		40.1 (38.0-42.2)		38.1 (37.3-39.0)
**Education, n (%)**									
	High school or under	73 (13.4)		11 (12.5)		8 (9)		92 (12.7)	
	College students or ungraduated	79 (14.5)		8 (9.1)		6 (6.7)		93 (12.9)	
	College graduate	294 (53.9)		48 (54.5)		47 (52.8)		389 (53.9)	
	Graduate student or master’s program	22 (4)		5 (5.7)		4 (4.5)		31 (4.3)	
	Master’s degree	63 (11.6)		15 (17)		22 (24.7)		100 (13.9)	
	Graduate student or doctoral program	4 (0.7)		0 (0)		2 (2.2)		6 (0.8)	
	Doctoral degree or PhD	10 (1.8)		1(1.1)		0 (0)		11 (1.5)	
**History of chronic diseases, n (%)**									
	No	510 (93.6)		70 (79.5)		77 (86.5)		657 (91)	
	Yes	35 (6.4)		18 (20.5)		12 (13.5)		65 (9)	
**BMI^c^, n (%)**									
	Underweight	41 (7.5)		5 (5.7)		3 (3.4)		49 (6.8)	
	Normal range	311 (57.1)		39 (44.3)		47 (52.8)		397 (55)	
	Overweight or obese	193 (35.4)		44 (50)		39 (43.8)		276 (38.2)	
BMI^c^, mean (95% CI)			22.8 (22.5-23.1)		24.2 (23.3-25.0)		23.9 (23.1-24.6)		23.1 (22.8-23.3)
**Waist measures, n (%)**									
	Unaware	468 (85.9)		66 (75)		57 (64)		591 (81.9)	
	Aware	77 (14.1)		22 (25)		32 (36)		131 (18.1)	
**Body fat measures, n (%)**									
	Unaware	499 (91.6)		71 (80.7)		69 (77.5)		639 (88.5)	
	Aware	46 (8.4)		17 (19.3)		20 (22.5)		83 (11.5)	
**Body weight self-interpretation, n (%)**									
	Incorrect	225 (41.3)		35 (39.8)		25 (28.1)		285 (39.5)	
	Correct	320 (58.7)		53 (60.2)		64 (71.9)		437 (60.5)	
**Stage of HEB^d^, n (%)^e^**									
	Precontemplation	124 (23)		17 (19.5)		14 (16.1)		155 (21.7)	
	Contemplation	268 (49.7)		29 (33.3)		28 (32.2)		325 (45.6)	
	Preparation	93 (17.3)		20 (23)		29 (33.3)		142 (19.9)	
	Action	25 (4.6)		9 (10.3)		3 (3.4)		37 (5.2)	
	Maintenance	29 (5.4)		12 (13.8)		13 (14.9)		54 (7.6)	
Stage of HEB, mean (95% CI)			2.20 (2.11-2.28)		2.66 (2.38-2.93)		2.69 (2.43-2.95)		2.31 (2.23-2.39)
**Stage of ALB^f^, n (%)^g^**									
	Precontemplation	131 (24.6)		18 (21.4)		13 (15.5)		162 (23.1)	
	Contemplation	263 (49.3)		28 (33.3)		27 (32.1)		318 (45.4)	
	Preparation	98 (18.4)		25 (29.8)		24 (28.6)		147 (21)	
	Action	19 (3.6)		5 (6)		6 (7.1)		30 (4.3)	
	Maintenance	22 (4.1)		8 (9.5)		14 (16.7)		44 (6.3)	
Stage of ALB, mean (95% CI)			2.13 (2.05-2.22)		2.49 (2.23-2.74)		2.77 (2.50-3.05)		2.25 (2.17-2.33)
**Use of health resources, n (%)**									
	No	456 (83.7)		58 (65.9)		34 (38.2)		548 (75.9)	
	Yes	89 (16.3)		30 (34.1)		55 (61.8)		174 (24.1)	

^a^E1: participants who had participated in web-based coaching for less than 1 year.

^b^E2: participants who had participated in web-based coaching for 1 year or more.

^c^The BMI classification used in Taiwan is as follows: underweight, BMI <18.5 kg/m^2^; normal weight, 18.5 kg/m^2^ ≤ BMI < 24 kg/m^2^; overweight, 24 kg/m^2^ ≤ BMI < 27 kg/m^2^; and obese, BMI ≥27 kg/m^2^.

^d^HEB: healthy eating behaviors.

^e^Control: n=539; E1: n=87; E2: n=87; and total: n=713.

^f^ALB: active living behaviors.

^g^Control: n=533; E1: n=84; E2: n=84; and total: n=701.

### Attention to Waist Circumstance and Body Fat Percentage

Most of the respondents did not know their waist circumference (591/722, 81.9%) or body fat percentage (639/722, 88.5%). However, the longer the duration of participation, the lower the percentage of unawareness. As shown in Table 1, a total of 85.9% (468/545) of the people in the control group did not know their waist circumference and 91.6% (499/545) of them did not know their body fat percentage. On the other hand, the corresponding estimates were 75% (66/88) and 81% (71/88) in group E1 and 64% (57/89) and 78% (69/89) in group E2.

The comparative statistics between the experimental groups E1 and E2 and the control group based on the generalized linear model are shown in Table 2. The odds of knowing their waist circumference were significantly higher (OR 2.12, 95% CI 1.24-3.63; *P*=.006) in group E2 than in the control group, as was the OR for body fat percentage (OR 2.30, 95% CI 1.24-4.29; *P*=.009). The OR for body fat percentage was also significantly higher in group E1 than in the control group (OR 2.31, 95% CI 1.21-4.43; *P*=.01).

**Table 2:** The long-term impacts on outcomes, analyzed by the generalized linear model.^a^

Variables and parameters		B		SE	95% Wald CI		Hypothesis testing				Exp(B) as odds ratio		95% Wald CI
							Wald chi-square (*df*)	*P* value					
**Waist measures awareness (yes or no)**													
	E1^b^	0.31	0.30		–0.27 to 0.89	1.11 (1)		.29	1.37			0.77 to 2.44	
	E2^c^	0.75	0.28		0.21 to 1.29	7.43 (1)		.006	2.12			1.24 to 3.63	
	Control	0^b^	—^d^		—	—		—	1			—	
**Body fat measures awareness (yes or no)**													
	E1^b^	0.84	0.33		0.19 to 1.49	6.42 (1)		.01	2.31			1.21 to 4.43	
	E2^c^	0.83	0.32		0.21 to 1.46	6.91 (1)		.009	2.30			1.24 to 4.29	
	Control	0^b^	—		—	—		—	1			—	
**Body weight self-interpretation (correct or incorrect)**													
	E1^b^	0.06	0.25		–0.42 to 0.55	0.06 (1)		.80	1.06			0.66 to 1.73	
	E2^c^	0.55	0.26		0.04 to 1.06	4.42 (1)		.04	1.73			1.04 to 2.89	
	Control	0^b^	—		—	—		—	1			—	
**Utilization of health resources** **(yes or no)**													
	E1^b^	0.71	0.27		0.18 to 1.25	6.74 (1)		.009	2.04			1.19 to 3.50	
	E2^c^	1.85	0.27		1.32 to 2.38	46.14 (1)		<.001	6.36			3.73 to 10.84	
	Control	0^b^	—		—	—		—	1			—	
**Stage of healthy eating behaviors^e^**													
	E1^b^	0.36	0.12		0.13 to 0.60	9.01 (1)		.003	—			—	
	E2^c^	0.35	0.12		0.11 to 0.59	8.38 (1)		.004	—			—	
**Stage of active living behaviors^e^**													
	E1^b^	0.29	0.12		0.06 to 0.53	5.85 (1)		.02	—			—	
	E2^c^	0.55	0.12		0.32 to 0.79	20.98 (1)		<.001	—			—	

^a^Covariates: sex, age, education level, employment status, history of chronic disease, BMI level.

^b^E1: participants who had participated in web-based coaching for less than 1 year.

^c^E2: participants who had participated in web-based coaching for 1 year or more.

^d^Not applicable.

^e^Compared with control group

### Correctness of Body Weight Self-Interpretation

Participants were asked to self-classify their current weight into 3 categories: below normal weight, normal weight, and above normal weight. The results showed that the longer the duration of participation, the higher the rate of correct classification or interpretation (control group: 320/545, 58.7%; group E1: 53/88, 60%; group E2: 64/89, 72%). In particular, the odds of correct classification of their own weight in group E2 was significantly higher (a near 2-fold increase) than that in the control group (OR 1.73, 95% CI 1.04-2.89; *P*=.04).

### Use of Health Resources

The question with regard to the use of health resources includes that of workout facilities and courses around the living environment, as well as that of various web-based health learning resources. The results show that only 24.1% (174/722) of the participants have used these resources. The longer the exposure to our program, the higher the rate of using these health-related resources (group E2: 55/89, 62%; group E1: 30/88, 34%; and control group: 89/545, 16.3%). Compared with the control group, both experimental groups (group E2: OR 6.36; *P*<.001; and group E1: OR 2.04; *P*=.009) showed significant greater chances (a 6-fold increase for group E2 and 2-fold increase for group E1) of using health resources.

### Behavior Stage Levels of Healthy Eating and Active Living

As shown in Table 2, the longer the period of exposure to our web-based program, the higher the reported behavioral stage level (as indicated by mean score) of implementing healthy eating and active living activities (see Table 1). Compared with the control group, the experimental groups E1 and E2 were significantly better in practicing healthy eating (group E1: *P*=.003; and group E2: *P*=.004) and active living (group E1: *P*=.02; and group E2: *P<*.001).

## Discussion

### Long-term Impact on Attention to Obesity-Related Measures or Indicators and the Ability to Interpret Them

Several recent national surveys have revealed that the rate of misperceived weight status among overweight or obese adults ranges from 40% to 77.5% [21-24]. Furthermore, the proportion of men who misperceive their weight status is significantly higher than that of women, and the surveys indicate that young people have a relatively low proportion of misperceived weight status [21-24].Post et al [43] conducted a brief survey with clinical patients and found that about 60% of the patients knew the correlation between BMI and obesity, but the vast majority of adult patients did not know the cut-off point of the normal range of BMI, and 70% of the patients were unaware their weight status and that they should be consulted by a physician.

The rate of misperceiving weight status in the control group of our study, the so-called “general public,” was approximately 41.3%. The 2 experimental groups, that is, those exposed to our web-based program, had significantly better weight status perception than the control group (Table 1). The longer the exposure, the higher the proportion of those who correctly interpreted the self-perceived body weight status. For those who had been exposed for over a year, their misinterpretation rate was as low as 28%. Furthermore, the experimental group with longer exposure time also showed greater attention to their waist circumference measures and body fat percentage than the control group. Compared with previous literature, our participants performed better in the proportion of those who correctly interpreted their body weight status [21]. This result suggests that the long-term web-based intervention strategy can indeed increase people’s attention to weight control parameter and gain knowledge on obesity definition, thus making correct judgment on one’s weight status.

### Long-term Impact on Practicing Healthy Living Behaviors

Some interventional studies showed that the intervention effect tends to peak in the sixth to eighth week, but at 20 to 36 weeks after the end of the intervention, researchers often find that the intervened health behaviors such as healthy eating and regular exercise would demonstrate a downward trend [26,31,37,44]. Our study used a web-based promotion strategy, which allowed us to interact with participants on a long-term basis. We found that the longer the participants were exposed to our program, the more advanced the behavioral stage with respect to healthy eating and active living (Table 2).

Moreover, the proportion of people who practiced HEB and ALB and stayed in the maintenance stage are much higher in those who participated for over a year (group E2) than those who participated for less a year (group E1) and the control group. The proportion of people who routinely used health-related resources almost doubled and increased 6-fold in those who had been exposed to our program for group E1 and group E2, respectively, compared to the control group (Table 1).

### Strengths and Limitations

This study not only provided long-term, stable, and readily available web-based learning resources, but it also designed interesting web-based activities with learning connotations and rewards. Participants in this study were able to join and actively participate through messages that were freely disseminated in the open web-based communities. Multiple benefits come with our intervention design that is primarily web-based and open to the public: (1) there is greater flexibility for people to freely participate and access the learning materials according to their own pace; (2) it is less likely to see the *waning effect* phenomenon of traditional short-term intervention courses, that is, once the course is over, the conditions and environment used to maintain the motivation of healthy lifestyle disappear; and (3) this study is conducting multiple outcome evaluations to verify the long-term interventional effect.

Although the results aligned with our anticipation, the research design of our web-based healthy behavior intervention through the WAKE.TAIWAN Facebook group may have its limitations. We cannot rule out the chance of selection bias since those who were actively participating are likely individuals with great intention to change their behavior [32]. In addition, although the self-administered questionnaire designed in this study adopts a factual question type design to minimize biases related to recall and context, a longer observation period is still required to validate the effectiveness of behavior change, weight control, and factors contributing to successful maintenance. Therefore, further follow-up investigations are needed to verify the intervention effect. On the other hand, an optimistic point of view is that our strategy can effectively recruit participants who are willing to change and may facilitate the process of strengthening healthy living behaviors. From the perspective of behavior change–related theories and relevant empirical results, providing sufficient supportive environment and resources for groups willing to accept changes will greatly help these participants to develop and maintain better healthy behaviors [29,31,36,37,44,45]. The results of this study echo this viewpoint.

In addition, our study used the Facebook learning group as the primary intervention channel, which could have caused a selection bias toward the Facebook users. The findings could not lend themselves directly to the use of other web-based tools or non–web-based strategies. Since a wide age range of public members and residents in urban and rural settings should be reached, digital division is an issue. For people with low digital condition, other supporting strategies must be considered.

### Conclusions

The results of this study confirmed that a long-term intervention strategy integrating web-based communities, audio and video channels, web-based learning resources, and web-based reward activities can improve adults’ attention to their own anthropometric measures and their ability to interpret self-perceived weight status while at the same time promoting the practice of healthy behaviors. Additional follow-up surveys are needed to examine whether this strategy can effectively assist people to control weight. Our research team is in the process of planning a follow-up survey in 2023. Changes in BMI, waist circumference, and body fat percentage will be longitudinally monitored for our program participants.

## Abbreviations

ALB: active living behaviors
CVI: content validity index
HEB: healthy eating behaviors
I-CVI: item-level content validity index
OR: odds ratio
S-CVI/UA: universal agreement scale-level content validity index

## Data Availability

The data sets generated during and/or analyzed during the current study are available from the corresponding author on reasonable request.
